# Some Experience with Dental Amalgams

**Published:** 1878-11

**Authors:** Henry S. Chase


### ARTICLE V.

### *Some Experience with Dental Amalgams.*

The tendency of practice to-day is towards *plastic* fillings. Of these, amalgamated metallic alloys hold an important place in Dentistry.

These alloys have been much improved in the last five years, and there are some of great value. The metals most usually employed are Tin and Silver. When composed of these alone, they do not make a plug which will retain its original shape for a long time; that is, there is a tendency to assume the *globular* shape, and hence, the plug draws away from the edges of the cavity, and leakage occurs. I have found silver and tin amalgams leak badly after six months, that did not leak at all the first four weeks.

What will prevent leakage? I find that a large proportion of gold will lessen the leakage in any formula of tin and silver. That in certain proportions of tin and silver the addition of gold will wholly prevent leakage, not only the first month but more than a year. My experience dates only for a period of two years. Antimony added to alloy in the same proportion, by *weight*, as gold, will prevent leakage as gold does. But as the bulk of an ounce of antimony is about three times that of an ounce of gold, it is easily seen that an oxidizable metal takes the place of one not so, in immense proportion. And, therefore, such an amalgam would not keep its color nearly as well as a gold alloy. Some very *leaky* amalgams keep a good color.

*Plasticity.*—Gold makes an amalgam more plastic.

An increase of tin makes an amalgam more plastic.

Antimony modifies plasticity.

An increase of mercury *prolongs* the period of plasticity.

*Hardness.*—An increase of silver makes an amalgam harder. The same is true of Antimony.

*Softness.*—An increase of tin makes a plug less hard. The same is true of gold.

*Brittleness.*—Antimony increases brittleness. The same is true of gold.

*Quick Setting.*—Silver promotes quick setting. Gold does the same.

Antimony retards it.

Tin retards it.

*Strength of Edge.*—Silver increases the strength or toughness of an amalgam.

*Color.*—A combination of metals which would keep a good color in one person's mouth might not in another, owing to the difference of gases and acids bathing the plug. A heavy proportion of gold in an alloy would cause the plug to keep bright more uniformly than others.

It is *not* the *mercury* that discolors, principally, in an amalgam, and therefore, so far as color is concerned, it matters not so much whether there is 30 or 100 per cent. of mercury.

The silver on the surface of an amalgam plug may be discolored by common salt forming argentic chloride. It may also be discolored by eating eggs, forming on its surface argentic sulphide. Both discolorations are easily removed by using water of ammonia. The tin on the surface of amalgam is affected in the same way as silver.

*Discolorations in Amalgamating an Alloy.*—When an amalgam is made of gold alone, no discoloration takes place, as may be proved in washing in alcohol. When *silver* alone is amalgamated, only as light yellow is perceived. When tin alone is amalgamated, a black oxide in great abundance is washed out. When an alloy of gold, silver and tin is amalgamated and washed, a great quantity of black oxide is washed out. A chemical analysis of this black wash shows it to be stannous oxide, or protoxide of tin.

The more gold there is in an amalgam, the more oxide of tin seems to be formed in the process of amalgamation. After this oxide is all washed out, an additional quantity of mercury to the clean mass will develop a fresh quantity of oxide, produced by the same means. If the alloy is amalgamated *under* alcohol, so that the air does not come to it, less oxide is formed.

Thus, we see that the oxidation is at the expense of the tin.

*Discoloration of the Surface of Amalgam Plugs.*—In mouths through which passes sulphuretted hydrogen, the tin and the silver may both be blackened, which black is stannous sulphide, and silver sulphide, and adheres tenaciously to the plug. The common fruit acids produce stannous oxide and silver suboxide, both black, but readily removed by the oral fluids.

The greater proportion of gold lessens oxidation and consequent discoloration.

*Discoloration of Dentos under Plugs.*—This is caused by leakage by the side of the walls, producing on the amalgam the black oxides already spoken of, which may show through a thin wall of dentos, or even a thick wall when the oxides penetrate the tubuli of the dentine.

*Oxide of Tin* (*Stannous Oxide.*)—If there is but little formed in amalgamation, it may be left in the mass for the posterior teeth.

A plug containing it, is lower on the scale of conductivity, and consequently is suitable for that portion of the cavity next to the pulp. On the other hand, it acts as a foreign substance, and as it makes the plug less dense, it is less strong, or is more brittle.

*Conservative Effects of Leakage.*—The stannous oxide that forms under a leaky plug and penetrates the dental tubuli, has a preservative effect on the dentos.

*Are Amalgams Stable.*—Those made of gold or silver or tin are permanent.

Amalgams of Sodium, Potassium or Aluminum are very unstable. Quicksilver will unite so quickly with aluminum that the heat created is sufficient to *burn* the palm of the hand on which it is mixed. It will then immediately crumble to powder. I tried aluminum in gold alloy by using only one part of aluminum to one hundred of the other metals, and found they would disintegrate in a short time. Even one-half per cent. of aluminum will ruin an amalgam after it is in the tooth. Mercury is held very tightly by silver. An amalgam containing silver 100 parts and mercury 43 parts refused to part with any of the latter metal, under a pressure of one hundred and fifty thousand pounds to the square inch! The ordinary dental amalgams will not part with any portion of their mercury, below 400 degrees of heat, if properly mixed.

The idea that amalgams are injurious to the system on account of the mercury they contain has been some time exploded.

Quick setting may be promoted by using warm instruments, but at the expense of strength in the plug.—*Dr. Henry S. Chase, in St. Louis Dental Quarterly.*

---

